# Supplementary material for: Therapeutic normal IgG intravenous immunoglobulin activates Wnt-β-catenin pathway in dendritic cells
Source: Commun Biol. 2020 Mar 4;3:96. doi: 10.1038/s42003-020-0825-4 (PMC7055225; doi:10.1038/s42003-020-0825-4)
Supplement: Supplementary file 1 — Description of Additional Supplementary Files [file 42003_2020_825_MOESM1_ESM.pdf]

Supplementary data 1 : Raw values of the experimental data presented in the article.
