# Supplementary material for: Therapeutic normal IgG intravenous immunoglobulin activates Wnt-β-catenin pathway in dendritic cells
Source: Commun Biol. 2020 Mar 4;3:96. doi: 10.1038/s42003-020-0825-4 (PMC7055225; doi:10.1038/s42003-020-0825-4)
Supplement: Supplementary file 3 — Supplementary Information [file 42003_2020_825_MOESM3_ESM.pdf]

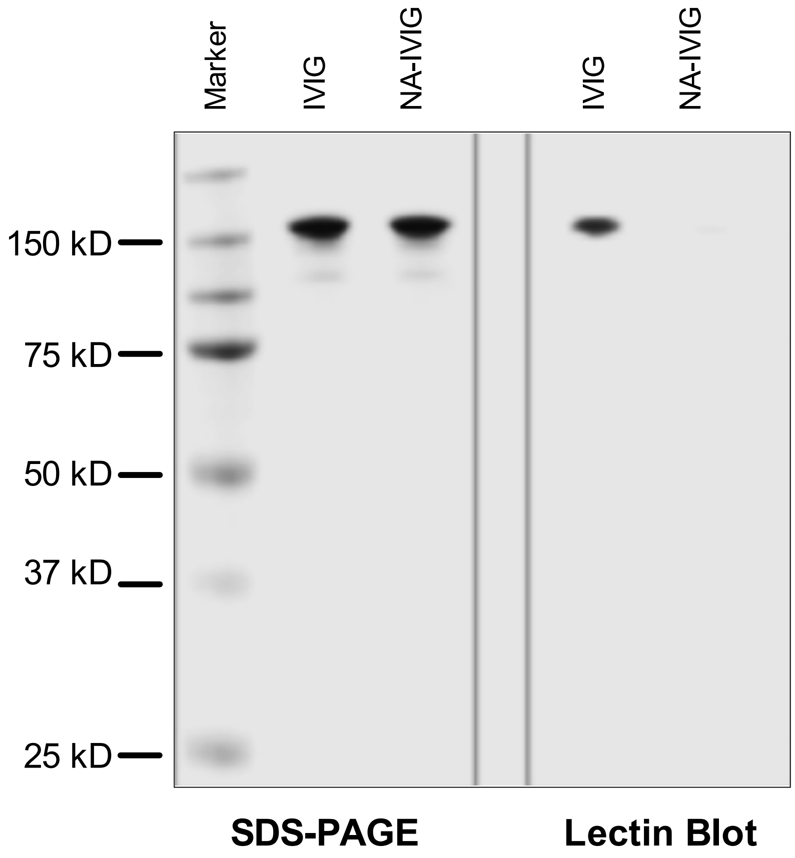

Supplementary Figure 1. SDS-PAGE (left) and lectin-blot (right) analyses of native and neuraminidase desialylated IVIG (NA-IVIG). Under non-reducing conditions, IVIG (1 µg) was subjected to SDS-PAGE. The gels were stained with Colloidal Coomassie (left) or transferred on to nitrocellulose, followed by incubation with biotin-SNA and AP-streptavidin. Blots were developed with chromogenic AP conjugate substrate (Right). The image is reproduced with modifications by permission from John Wiley and Sons, Inc. (Ref.[38]).

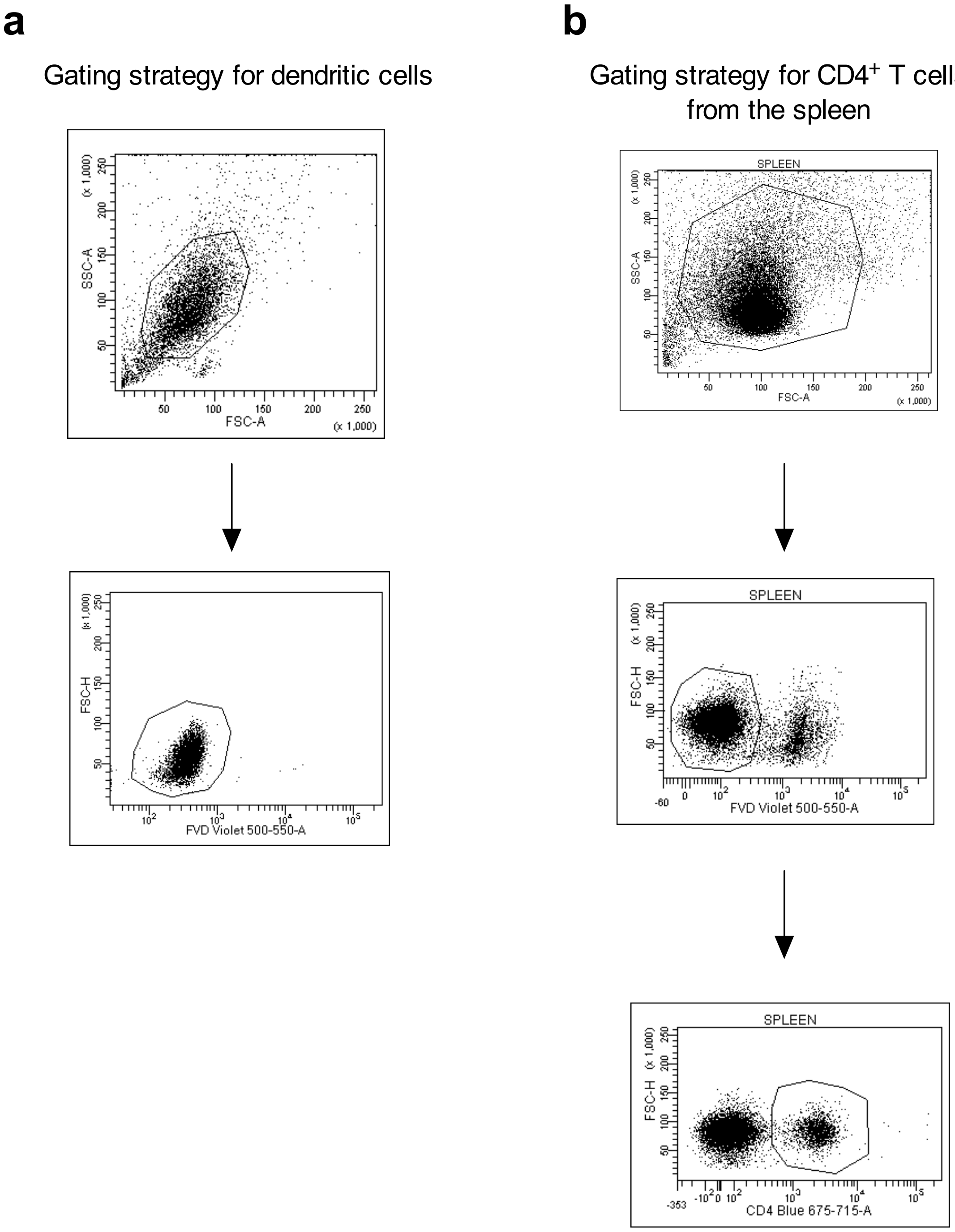

Supplementary Figure 2. The flow cytometer gating strategy for **(a)** human dendritic cells and **(b)** $CD4^{+}$ T cells from the mouse spleen.

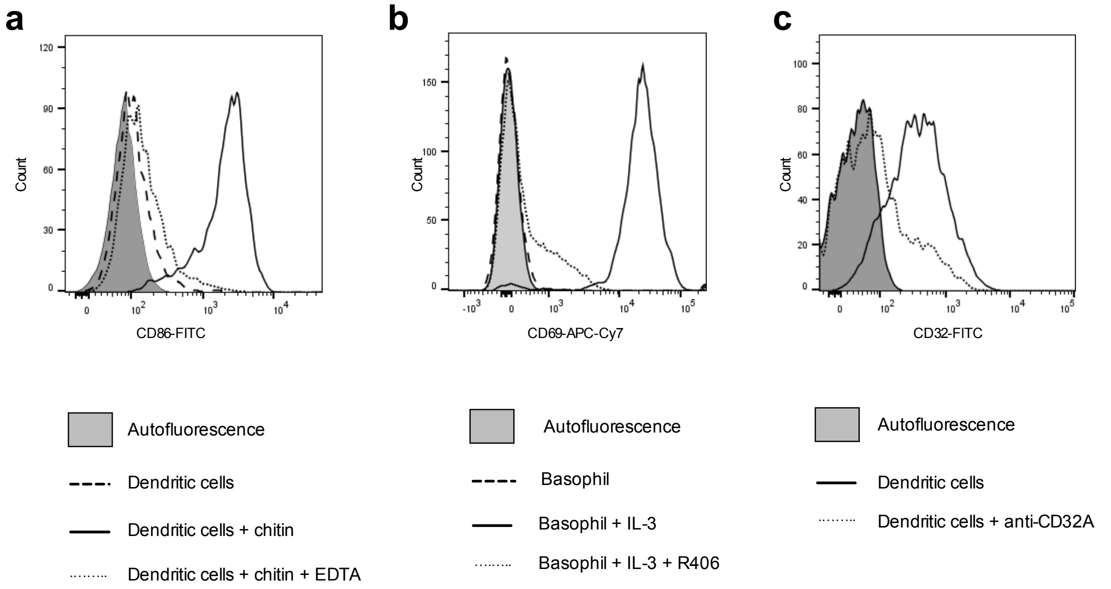

Supplementary Figure 3. The assays for the efficacy of ethylenediaminetetraacetic acid (EDTA), Syk inhibitor R406 and blocking monoclonal antibody (MAb) to FcγRIIA. **(a)** dendritic cells (DCs, 0.5 million cells/ml) were pretreated with EDTA (0.5 mM) for one hour before stimulating with chitin polysaccharide for 24 hours. The expression of CD86 was analyzed by flow cytometry. **(b)** Peripheral blood basophils (0.1 million/ 200 µl) were incubated with Syk inhibitor, R406 (10 µM) for 1 hour followed by stimulation with IL-3 (20 ng/200 µl) for 24 hours. The expression of CD69 was analyzed by flow cytometry. **(c)** DCs (0.5 million cells/ml) were pre-incubated with blocking MAb (clone IV.3) to human FcγRIIA (10 µg/0.5 million cells) for one hour at 4°C. The cells were then incubated for 30 minutes with FITC-conjugated anti-human CD32 MAb (Clone FLI8.26) that detects CD32 irrespective of isoforms. The expression of CD32 was analyzed by flow cytometry.

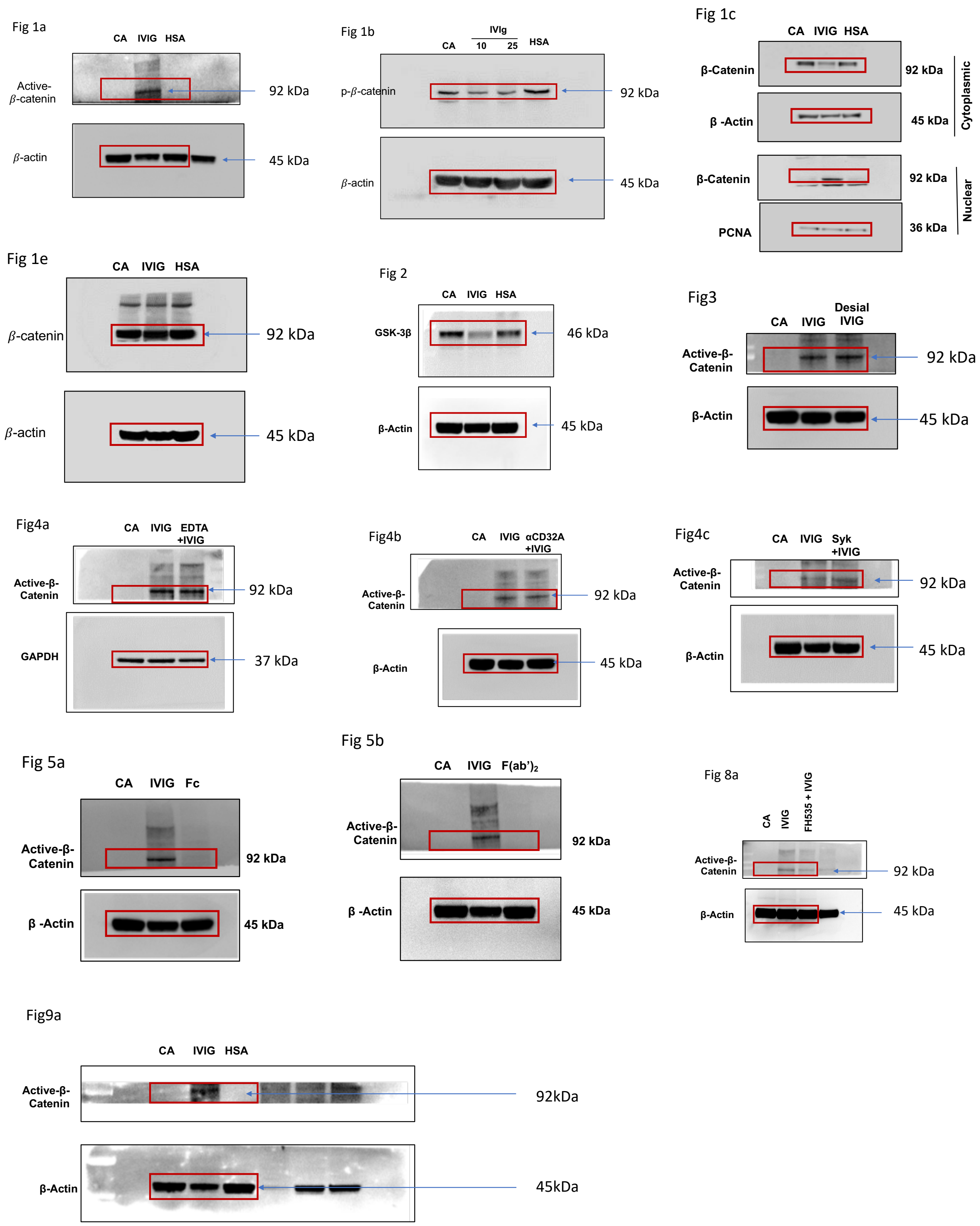

Supplementary Figure 4. The full western blot images. The western blot images that are shown in the article are highlighted by boxes.
