# Supplementary material for: Therapeutic normal IgG intravenous immunoglobulin activates Wnt-β-catenin pathway in dendritic cells
Source: Commun Biol. 2020 Mar 4;3:96. doi: 10.1038/s42003-020-0825-4 (PMC7055225; doi:10.1038/s42003-020-0825-4)
Supplement: Supplementary file 5 — Reporting Summary [file 42003_2020_825_MOESM5_ESM.pdf]

nature research

Corresponding author(s): Jagadeesh BAYRY

Last updated by author(s): Jan 22, 2020

Reporting Summary

Nature Research wishes to improve the reproducibility of the work that we publish. This form provides structure for consistency and transparency in reporting. For further information on Nature Research policies, see Authors & Referees and the Editorial Policy Checklist.

Statistics

For all statistical analyses, confirm that the following items are present in the figure legend, table legend, main text, or Methods section.

n/a	Confirmed	
☐	☒	The exact sample size (n) for each experimental group/condition, given as a discrete number and unit of measurement
☐	☒	A statement on whether measurements were taken from distinct samples or whether the same sample was measured repeatedly
☐	☒	The statistical test(s) used AND whether they are one- or two-sided<br>*Only common tests should be described solely by name; describe more complex techniques in the Methods section.*
☒	☐	A description of all covariates tested
☐	☒	A description of any assumptions or corrections, such as tests of normality and adjustment for multiple comparisons
☐	☒	A full description of the statistical parameters including central tendency (e.g. means) or other basic estimates (e.g. regression coefficient) AND variation (e.g. standard deviation) or associated estimates of uncertainty (e.g. confidence intervals)
☐	☒	For null hypothesis testing, the test statistic (e.g. F, t, r) with confidence intervals, effect sizes, degrees of freedom and P value noted<br>*Give P values as exact values whenever suitable.*
☒	☐	For Bayesian analysis, information on the choice of priors and Markov chain Monte Carlo settings
☒	☐	For hierarchical and complex designs, identification of the appropriate level for tests and full reporting of outcomes
☒	☐	Estimates of effect sizes (e.g. Cohen's d, Pearson's r), indicating how they were calculated

Our web collection on statistics for biologists contains articles on many of the points above.

Software and code

Policy information about availability of computer code

Data collection	BD FACS DIVA for flow cytometry
Data analysis	GraphPad Prism, BD FACS DIVA, Microoift Excel, myImageAnalysis software v 2.0

For manuscripts utilizing custom algorithms or software that are central to the research but not yet described in published literature, software must be made available to editors/reviewers. We strongly encourage code deposition in a community repository (e.g. GitHub). See the Nature Research guidelines for submitting code & software for further information.

Data

Policy information about availability of data

All manuscripts must include a data availability statement. This statement should provide the following information, where applicable:

- Accession codes, unique identifiers, or web links for publicly available datasets
- A list of figures that have associated raw data
- A description of any restrictions on data availability

All data are included in this article and figures. No restrictions on the data availiability

Field-specific reporting

Please select the one below that is the best fit for your research. If you are not sure, read the appropriate sections before making your selection.

☒ Life sciences ☐ Behavioural & social sciences ☐ Ecological, evolutionary & environmental sciences

For a reference copy of the document with all sections, see nature.com/documents/nr-reporting-summary-flat.pdf

Life sciences study design

All studies must disclose on these points even when the disclosure is negative.

Sample size	Sample sizes of three to seven per group were chosen for in vitro experiments using human immune cells and for in vivo experiments, ten mice per group was chosen as this would allow the detection of a 25% difference in the mean between experimental and control groups with a probability of greater than 95% ($p < 0.05$). Acquisition of data from multiple independent donors allowed us to control inter-individual variations.
Data exclusions	Only data from the experiments with technical errors (such as unsuccessful stimulation or staining) or unsatisfactory quality of buffy bags and cells , non-specific death of mice early during induction of EAE were excluded from the analysis.
Replication	Replication is ensured by the use of several independent donors and mice experiments
Randomization	The mice were randomly grouped before the induction of EAE. For in vitro experiments, there was no intervention. Hence, randomization was not used.
Blinding	Blinding was not used

Reporting for specific materials, systems and methods

We require information from authors about some types of materials, experimental systems and methods used in many studies. Here, indicate whether each material, system or method listed is relevant to your study. If you are not sure if a list item applies to your research, read the appropriate section before selecting a response.

Materials & experimental systems

n/a	Involved in the study	
☐	☒	Antibodies
☒	☐	Eukaryotic cell lines
☒	☐	Palaeontology
☐	☒	Animals and other organisms
☐	☒	Human research participants
☒	☐	Clinical data

Methods

n/a	Involved in the study	
☒	☐	ChIP-seq
☐	☒	Flow cytometry
☒	☐	MRI-based neuroimaging

Antibodies

Antibodies used	For immunoblots following antibodies were used: Rabbit MAbs to non-phospho (active) β-catenin (Ser33/37/Thr41) (clone D13A1), GSK-3β (clone 27C10), GAPDH (14C10), β-Actin (HRP-conjugated) (clone 13E5); polyclonal rabbit antibodies to phospho-β-catenin (Ser33/37/Thr41) (#9561) and β-catenin (#9562) were from Cell Signaling Technology (Ozyme, Saint Quentin Yvelines, France). HRP-conjugated human adsorbed goat anti-rabbit IgG secondary antibody (#4010-05) was purchased from Southern Biotech (Birmingham, LA).<br><br>For flow cytometry, the following antibodies were used: FITC-conjugated anti-mouse MAbs to IFN-γ (clone XMG1.2) and CD25 (clone 7D4), anti-human CD32 (Clone FLI8.26); PE-conjugated anti-mouse MAb to IL-17A (clone TC11-18H10); Per-CP/Cy5.5-conjugated anti-mouse MAb to CD4 (clone RM4-5); APC/Cy7-conjugated anti-human MAb to CD69 (Clone FN50)were from BD Biosciences (Le Pont de Claix, France). APC-conjugated MAb to FOXP3 (clone FKJ-16s), APC-conjugated anti-human HLA-DR (clone G46-6) and Fixable Viability Dye eFlour506 were from eBioscience (Paris, France). Alexa Flour 488-conjugated goat anti-rabbit IgG (H+L) was from Invitrogen (ThermoFisher Scientific, Illkirch , France #A11034).<br><br>For functional assay the following antibody was used: Blocking MAbs to FcγRIIA (clone IV.3) was purchased from Stem Cell Technologies (Grenoble, France).
Validation	Flow-cytometry and immunoblot antibodies were validated by the manufacturers. Also flow-cytometry antibodies were validated in the lab for previous reports (For example: Othy et al. J Immunol 2013; Trinath et al. Blood 2013; Othy et al. Eur J Immunol 2014; Stephen-Victor et al. J Infect Dis 2017; Galeotti et al. J Allergy Clin Immunol 2019)

Animals and other organisms

Policy information about studies involving animals; ARRIVE guidelines recommended for reporting animal research

Laboratory animals	Eight-week old, C57BL/6J female mice
Wild animals	Not applicable

Field-collected samples	Not applicable
Ethics oversight	Ethical committee for animal experimentation and French Ministry of Higher Education and Research (APAFIS#10539-2017070715163055 V4)

Note that full information on the approval of the study protocol must also be provided in the manuscript.

Human research participants

Policy information about studies involving human research participants

Population characteristics	Buffy bags of healthy donors were purchased from the Centre Necker-Cabanel, EFS, Paris. The identity of the donors are blinded at purchase
Recruitment	Not applicable
Ethics oversight	L'Établissement Français du Sang, Paris- INSERM, Paris ; ethical committee permission 15/EFS/012; 18/EFS/033

Note that full information on the approval of the study protocol must also be provided in the manuscript.

Flow Cytometry

Plots

Confirm that:

- [x] The axis labels state the marker and fluorochrome used (e.g. CD4-FITC).
- [x] The axis scales are clearly visible. Include numbers along axes only for bottom left plot of group (a 'group' is an analysis of identical markers).
- [x] All plots are contour plots with outliers or pseudocolor plots.
- [x] A numerical value for number of cells or percentage (with statistics) is provided.

Methodology

Sample preparation	Peripheral blood monocytes were obtained from buffy bags of healthy donors and differentiated to DCs as previously detailed. DC were cultured in 12 well plate at 0.5x106 cells/0.5 ml of Accell Delivery media. 1µM of nontargeting control siRNA or LRP5 and LRP6 siRNA (Dharmacon; Thermo Fisher Scientific) were introduced into cells for 72 hours. After 72 hours, cells were cultured in RPMI with 10% FCS and IVIG for 24 hours followed by FACS analysis. Surface staining of DCs was performed with fluorescence-conjugated MAb HLA-DR. For active-β-catenin detection, cells were stained with rabbit MAbs to non-phospho (active) β-catenin (Ser33/37/Thr41) and followed by Alexa Flour 488-conjugated goat anti-rabbit IgG (H+L) by using Cell Signaling Buffer Set A (Miltenyi Biotec).<br><br>Splenocytes were isolated and RBC were lysed using ACK lysis buffer. 0.5 million cells/ml were stimulated with phorbol myristate acetate (PMA) (50 ng/ml/0.5 million cells) and ionomycin (500 ng/ml/0.5 million cells), along with GolgiStop for 4 hours. Th1, Th17, and Treg populations were analyzed by combination of surface and intracellular staining for various markers. Surface staining was performed with fluorescence-conjugated MAbs to CD4 and CD25. After fixation and permeabilization by intracellular staining kit (eBioscience), intracellular staining with fluorescence-conjugated MAbs to IFN-γ, IL-17A and FOXP3 were carried out. Samples were acquired using LSR-II flow cytometer (BD Biosciences) and data were analyzed by FACS-DVIA (BD Biosciences).<br><br>For flow cytometry, the following antibodies were used: FITC-conjugated anti-mouse MAbs to IFN-γ (clone XMG1.2) and CD25 (clone 7D4), anti-human CD32 (Clone FLI8.26); PE-conjugated anti-mouse MAb to IL-17A (clone TC11-18H10); Per-CP/Cy5.5-conjugated anti-mouse MAb to CD4 (clone RM4-5); APC/Cy7-conjugated anti-human MAb to CD69 (Clone FN50)were from BD Biosciences (Le Pont de Claix, France). APC-conjugated MAb to FOXP3 (clone FKJ-16s), APC-conjugated anti-human HLA-DR (clone G46-6) and Fixable Viability Dye eFlour506 were from eBioscience (Paris, France). Alexa Flour 488-conjugated goat anti-rabbit IgG (H+L) was from Invitrogen (ThermoFisher Scientific, Illkirch , France #A11034).
Instrument	LSR II (BD Biosciences)
Software	FACSDiva and FlowJo
Cell population abundance	MicroBeads from Miltenyi Biotec (CD14) were used for isolating monocytes from PBMC.<br>As pure dendritic cell population was used, cells were directly analyzed on live cells.<br>For CD4 T cell response in mice splenocytes, CD4 cells were first gated on live cell population and were then analyzed for various T cell subsets.

Gating strategy

Initial gating using FSC/SSC plot to select cells and exclude debris, followed by selection of viable cells using fixable viability dye eFluor 506.
As pure dendritic cell population was used, surface markers were analyzed on live cells.
For CD4 T cell response in mice splenocytes, CD4 cells were first gated on live cell population and were then analyzed for various T cell subsets.

☒ Tick this box to confirm that a figure exemplifying the gating strategy is provided in the Supplementary Information.
